# Association between treatment outcomes and extent of chest X-ray involvement among pulmonary tuberculosis patients under RNTCP in North India: A prospective study

**DOI:** 10.6026/973206300214448

**Published:** 2025-12-15

**Authors:** Mahesh Kumar Patidar, Rakesh Kumar Sisodia, Dhiresh Jaiswal, Shushil Munjal, Deepak Nagar

**Affiliations:** 1 Department of Respiratory Medicine, Government Medical College, Ratlam, Madhya Pradesh, India; 2 Department of General Medicine, Government Medical College, Ratlam, Madhya Pradesh, India; 3 Department of Respiratory Medicine, Government Medical College, Datia, Madhya Pradesh, India; 4 Department of Respiratory Medicine, National Institute of Tuberculosis and Respiratory Diseases (NITRD), New Delhi, India

**Keywords:** Pulmonary Tuberculosis, drug-sensitive, chest X-ray

## Abstract

Tuberculosis (TB) remains one of the leading causes of morbidity and mortality in India, with pulmonary involvement contributing significantly to disease burden and transmission. Hence, this prospective observational study enrolled new and previously treated sputum smear-positive patients from the NITRD DOTS area 1st September 2019 to 31st October 2019. All participants received a 6-month drug-sensitive TB regimen (2 months of HRZE followed by 4 months of HRE) to assess treatment outcomes at the end of six months. The study also analyzed whether the extent of chest X-ray involvement was associated with treatment outcome. Data shows that both the extent of chest X-ray involvement and the presence of cavities on chest X-ray were found to be significantly associated with unsuccessful treatment outcomes.

## Background:

Approximately 10 million individuals get TB annually on a global scale. Not only is tuberculosis (TB) one of the top ten killers, but it also ranks higher than HIV/AIDS as the major infectious agent causing tuberculosis. Though tuberculosis may strike anybody at any time, the vast majority of cases occur in adults (about 90%). The male-to-female ratio is 2:1, and the annual incidence rate varies throughout the country from under 50 to over 5,000 per 1 million people. Countries with a high tuberculosis burden account for over 90% of annual cases. About 1.7 billion individuals throughout the world have M. tuberculosis infections, putting them at risk of contracting the illness [1]. The 2019 Global TB report estimates that there were over 26.9 lakh cases of tuberculosis in India. There were 87% of newly diagnosed tuberculosis patients and 13% of retreatment cases in India [2]. Notified tuberculosis (TB) patients, including those in the private sector, are now eligible for a universal drug susceptibility test (DST) to determine if they are resistant to rifampicin when they are diagnosed, in accordance with existing policy [3]. Previously for newly diagnosed cases of PTB CAT 1 (2HRZE/4HR) regimen was given and retreatment cases of TB received CAT 2 (1 HRZES/2HRZE/5HRE) regimen. But from Dec 2018 Government of India on expert advice started CAT 1 regimen for retreatment cases of PTB. Several factors lead to the discontinuation of the CAT 2 regimen. As per WHO REPORT on TB 2017 CAT 2 regimen had several problems. The current gold standard in patient care is to determine the patient's medication resistance profile and treat them appropriately after a drug susceptibility test has been administered to everyone who has had a treatment interruption or illness relapse [4]. Impunity in treatment across patients, postponement of effective treatment for drug-resistant tuberculosis (a practice that worsens community and patient outcomes), and needless exposure to streptomycin toxicity in patients with drug-susceptible disease would result from a lack of drug-susceptibility testing and the use of substandard category II regimens in empirical treatment. An important tenet of tuberculosis therapy is that an ineffective regimen should not be supplemented with a single additional medicine [5]. Streptomycin is no longer used as a second-line agent in multidrug-resistant tuberculosis (MDR) treatment because it is added to an already ineffective regimen that includes isoniazid, rifampicin, ethambutol, and pyrazinamide, which goes against this concept and promotes the development of drug resistance. Compared to newly diagnosed tuberculosis patients, individuals with a history of drug resistance are more likely to have discontinued first-line TB therapy or had disease recurrence. This fundamental therapeutic concept is at odds with using a category II regimen on these patients, which would hasten the development of medication resistance [6]. Therefore, it is of interest to compare the treatment success rate in newly diagnosed and previously treated PTB cases.

## Materials and Methodology:

## Study area:

Study was conducted in field condition in NITRD DOTS area (Ladosarai, Mehrauli, Chhatarpur, Khanpur, Tigri) New Delhi.

## Study period:

Study period from 1st September 2019 to 31 March 2020. Patient's enrolment was done from 1st September to 31st October and then follows up till end of treatment.

## Study design:

The study was prospective Observational

## Study population and sample size:

When we look at data from the previous year from there five DOTS centers of NITRD, approx. 8-10 sputum smear positive patients per month in each DOTS area of NITRD were diagnosed as sputum positive PTB (new and previously treated). As we enrolled patient from 1st September to 31st October (2 month)). So, the on the basis of these previous year data our sample size were 100 patients.

## Inclusion Criteria:

All newly diagnosed and previously treated sputum smear positive pulmonary tuberculosis patient who are registered under revised national tuberculosis control programme (RNTCP) in NITRD DORS AREA.

## Exclusion Criteria:

[1] All drug resistant (INH and RMP) cases of pulmonary Tuberculosis.

[2] Refusal to participate in study.

## Methodology:

Patient was enrolled in study that full fill the inclusion criteria. Patients were enrolled from five DOTS area of NITRD (Lado Sarai, Chhatarpur, Khanpur, Tigre, Mehrauli, New Delhi) for study. Sputum smear Microscopy, CBNAAT, LPA and drug sensitivity testing were carried out in all enrolled cases of PTB to look for drug resistant. We enrolled only sputum smear positive drug sensitive PTB patients. At the conclusion of the intense phase and again at the conclusion of therapy, patients were questioned twice to assess their progress and outcomes.

##  Treatment outcome definitions as per PMDT 2019:

## Cured:

The patient must have been microbiologically verified at treatment initiation and must have been smear negative at treatment completion and on a prior occasion.

## Treatment completed:

A patient who, after following all prescribed treatment plans, nevertheless does not have a positive microbiological test and is thus considered to have failed therapy or not cured.

## Treatment failed:

An individual whose blood sample tests positive at 5 months or later.

## Died:

Person who has passed away while receiving anti-TB therapy, for any cause.

## Lost to follow up:

An individual whose therapy has been continuously stopped for a month or more.

## Note evaluated:

A patient who has not been allocated a treatment result. "still on treatment" and "transfer-out" have both been included in the past.

## Regimen changed:

Before a regimen may be considered a failure, it must be permanently discontinued and replaced with a new one that includes a change in at least one anti-TB medicine.

## Ethical consideration:

For all cases, informed consent in English and Hindi were taken after thoroughly explaining them about the study. Only patients giving consent were enrolled in the study. There was no financial burden to the patients for participation in the study.

## Data entry and statistical analysis:

After data was obtained, it was coded and put into Microsoft Excel as variables. The SPSS-PC-20 version was used for statistical analysis and evaluation of the data. A chi-square test or fisher exact test was used to assess for statistical differences between the proportions, whilst qualitative data were reported as percentages and quantitative data as mean ± standard deviation. Statistical significance was defined as a "P" value below 0.05.

## Results:

The mean age± standard deviation of our study sample was 33.52±15.90 years respectively. When classified according to age the maximum patients 39(39%) belonged to age group 11-25 years, followed by 32 patients belongs to age group 26-40 years ,16 patients belonged to age group 41-55 years,12 patients belonged to age group 56-70 years, 1 patient belonged to age group >70 years (Table 1 - see PDF, Figure 1 - see PDF). In our study, out of enrolled 100 patients 60 (60%) were males 40 (40%) were females (Table 2 - see PDF, Figure 2 - see PDF). Among newly diagnosed patients (n=77) outcome of 73(94.8%) patients were successful and outcome of 4(5.2%) patients were unsuccessful. Out of previously treated patients (n=23) outcome of 19(82.6%) patients were successful while outcome of 4(17.4%) patients were unsuccessful. we found no statistically significant association between the outcome between new and previously treated PTB patients (P value=0.07) (Table 3 - see PDF, Figure 3 - see PDF). Among 100 enrolled patients 32(32%) patient had cavity in their chest x ray and 68(68%) patients had no cavity in their chest x ray out of 32 patients 25(78.1%) patients had successful outcome while 7(21.9%) patients had unsuccessful outcome among 68(68%) patients who had no cavity in their chest x ray, 67(98.5%) patients had successful outcome and 1(1.5%) patients had unsuccessful outcome. In our study we found statistically significant relation between presence of cavity in chest x ray with unfavourable outcome in PTB (P value =<0.001) (Table 4 - see PDF, Figure 4 - see PDF). Among 100 enrolled participants 65% patient had non extensive chest x ray involvement and 35 % patients had extensive involvement of chest x ray, among (n=65) 65% patients, 63(96.9%) patients had successful outcome while 2 (3.1%) patients had unsuccessful outcome. Among (n=35) 35 % patients, 29 (82.9%) patients had successful outcome while 6 (17.1%) patients had unsuccessful outcome. It was analyzed that statistically significant association of extensive involvement of chest x ray with unfavourable outcome (P value=0.002) (Table 5 - see PDF, Figure 5 - see PDF). When analyzed by age, treatment success was observed in 92.9% of patients aged ≤40 years and 89.7% of those aged >40 years. The difference was not statistically significant (p = 0.70), suggesting that age did not play a major role in determining treatment success within this cohort. Similarly, gender did not influence outcome: 90% of males and 95% of females had successful results (p = 0.47), indicating comparable response to therapy across sexes (Table 6 - see PDF).

## Discussion:

The present study was undertaken to compare the treatment success rate of Category I antitubercular therapy (ATT) among newly diagnosed and retreatment pulmonary tuberculosis (PTB) cases under the National Tuberculosis Elimination Programme (NTEP) at NITRD, New Delhi. The results highlight a high overall treatment success rate, reflecting the effectiveness of the current drug-sensitive regimen (2HRZE/4HRE) and the improved implementation of directly observed treatment strategies at the community level. The demographic distribution of patients indicated that tuberculosis predominantly affects individuals in their most productive age group, with a higher proportion of male patients. This pattern suggests both biological susceptibility and socio-behavioral determinants contributing to disease prevalence in younger and working-age populations. The male predominance may be attributed to increased exposure to infection sources, occupational stress, and higher rates of smoking and alcohol use, which are established risk factors for tuberculosis progression and relapse [2]. Radiological findings played a crucial role in determining prognosis. The presence of cavitation on chest radiographs was found to be significantly associated with unsuccessful outcomes. Cavities often represent a higher bacillary load, delayed sputum conversion, and prolonged infectivity, all of which contribute to treatment failure or relapse. Similarly, extensive radiological involvement was correlated with unfavourable outcomes, indicating a higher disease burden and delayed resolution with standard therapy [5]. These findings underscore the importance of early diagnosis and timely initiation of treatment to prevent extensive lung damage and improve treatment success. The comparison between new and previously treated cases revealed comparable outcomes under the current regimen. The lack of significant difference in success rates between these groups reflects the effectiveness of the updated drug-sensitive regimen and the practice of ruling out drug resistance at baseline using molecular diagnostic tools. This approach ensures appropriate categorization and prevents the inadvertent use of ineffective regimens in resistant cases [7, 8- 9]. The overall success rate of 93% observed in this study is notably higher than previously reported national averages. This improvement can be attributed to enhanced adherence monitoring, standardized drug supply, increased patient education, and improved access to diagnostic facilities under the NTEP framework [10, 11-12]. The low rates of failure and loss to follow-up further emphasize the robustness of the directly observed treatment system and the strengthened follow-up mechanisms at DOTS centers [13-14]. The identification of radiological predictors of poor outcomes-such as cavitary and extensive lesions-has important clinical implications. These patients may require intensified monitoring, extended therapy, or adjunctive nutritional and psychosocial support to ensure treatment completion and reduce relapse risk. Additionally, comorbid conditions such as diabetes, malnutrition, and substance use, though not extensively analyzed in this study, could further modulate treatment outcomes and warrant detailed assessment in future research [15, 16-17]. In summary, the findings of this study reaffirm that the revised Category I regimen under NTEP is highly effective for both new and retreatment cases of drug-sensitive pulmonary tuberculosis when implemented with proper diagnostic stratification, adherence monitoring, and radiological follow-up. The continued emphasis on individualized patient support, early detection of resistance, and public awareness remains essential for sustaining high cure rates and achieving the national goal of tuberculosis elimination.

## Conclusion:

In this study of 100 patients, 60 (60%) were male and 40 (40%) were female. The mean age ± standard deviation of the study sample was 33.52 ± 15.90 years. When classified by age, the largest group of patients, 39 (39%), belonged to the 11-25 years age bracket, followed by 32 patients in the 26-40 years age group. Thus, both the extent of chest X-ray involvement and the presence of cavities on chest X-ray were significantly associated with unsuccessful treatment outcomes.

## References

[1] Dholakia YN (2012). Infect Dis Rep..

[2] Ying C (2022). Int J Infect Dis..

[3] Chatterjee A (2010). J Clin Microbiol..

[4] https://www.ncbi.nlm.nih.gov/books/NBK92617/.

[5] Wáng YXJ (2018). Quant Imaging Med Surg..

[6] Huang Q (2016). Eur J Med Res..

[7] Singh AV (2021). BMC Microbiol..

[8] Gopalan N (2021). PLoS One..

[9] Semunigus T (2016). Ann Clin Microbiol Antimicrob..

[10] Grozdanovic Z (2015). PLoS One..

[11] Bommart S (2021). Diagn Interv Imaging..

[12] Sumana M (2024). Journal of Family Medicine and Primary Care..

[13] Mistry N (2020). BMJ Open..

[14] Tonne EO (2022). Eur J Radiol Open..

[15] Lilhore S (2025). International Journal of Science and Research (IJSR)..

[16] Rastoder E (2019). Clin Tuberc Other Mycobact Dis..

[17] Sato R (2025). BMC Infect Dis..

